# Incidence and risk factors for first line anti retroviral treatment failure among Ugandan children attending an urban HIV clinic

**DOI:** 10.1186/1742-6405-10-25

**Published:** 2013-11-11

**Authors:** Robert Sebunya, Victor Musiime, Sabrina Bakeera Kitaka, Grace Ndeezi

**Affiliations:** 1 Department of Paediatrics and child Health Makerere, University college of Health Sciences, Kampala, Uganda; 2 Joint Clinical Research Centre, Kampala, Uganda

## Abstract

**Background:**

Early recognition of antiretroviral therapy (ART) failure in resource limited settings is a challenge given the limited laboratory facilities and trained personnel. This study aimed at describing the incidence, risk factors and the resistance associated mutations (RAMs) of first line treatment failure among HIV-1-infected children attending the Joint Clinical Research Centre (JCRC), Kampala, Uganda.

**Methods:**

A retrospective cohort of 701 children who had been initiated on ART between January 2004 and September 2009 at the JCRC was studied. Data of children aged 6 months up to 18 years who had been started on ART for at least 6 months was extracted from the clinic charts. The children who failed the first-line ART were taken as cases and those who did not fail as the controls. Data was analysed using STATA version10.

**Results:**

Of 701 children, 240(34%) failed on first line ART (cases) and 461(66%) did not fail (controls). The overall median time (IQR) to first line ART failure was 26.4 (18.9 – 39.1) months. The factors associated with treatment failure were poor adherence [(OR = 10, 95 CI: 6.4 – 16.7) p < 0.001], exposure to single dose nevirapine (sdNVP) [(OR = 4.2, 95% CI:1.8-9.4), p = 0.005] and a NVP containing regimen [(OR = 2.2,95% CI:1.4-3.6), p < 0.001]. Of 109 genotypic resistance profiles analyzed, the commonest non nucleoside reverse transcriptase inhibitor (NNRTI) resistance associated mutations (RAM) were: K103N (59; 54%)), Y181C (36; 27%)) and G190A (26; 24%)) while the commonest nucleoside reverse transcriptase inhibitor (NRTI) RAM was the M184V (89; 81%). Thymidine analogue- mutations (TAMs) were detected in 20% of patients.

**Conclusions:**

One in three children on first-line ART are likely to develop virological treatment failure after the first 24 months of therapy. Poor adherence to ART, a NVP based first-line regimen, prior exposure to sdNVP were associated with treatment failure.

## Introduction

Antiretroviral therapy (ART) has been shown to reduce human immunodeficiency virus (HIV) associated morbidity and mortality by restoring and preserving the immunological function [1-6]. Globally, there has been a pronounced increase in scaling up ART services notably in sub-Saharan Africa [7,8], with Uganda being among the pioneering countries [9]. However, ART is long term treatment with the potential of drug toxicity and probable emergency of resistance which results into treatment failure. Treatment failure is suboptimal response or a lack of sustained response to therapy using either clinical, immunological and virological criteria [10]. Paediatric antiretroviral therapy cohorts in low income countries have reported that response to ART is as good or comparable to that in high income countries [4,11]. Single dose nevirapine (sNVP) has been widely used for prevention of mother to child transmission (PMTC) in low resource settings and NVP continues to be used as part of the backbone of non-nucleoside reverse transcriptase inhibitors (NNRTI) among HIV infected children [12]. Some of these cohorts have reported occurrence of resistant mutations to sNVP with subsequent treatment failure [13,14]. The incidence and risk factor for first line ART has not been described in Uganda. In this study we describe the incidence, risk factors for the first line treatment failure among children attending one of the largest paediatric ART program in Uganda. We also describe the resistance associated mutations in this cohort.

Whereas viral load is the ideal tool for monitoring ART response, clinical and immunological monitoring are widely used in resource limited settings like Uganda. There is sufficient evidence suggesting a poor correlation between clinical/immunological and virological ART failure [15-17]. In Uganda like many other sub-Saharan countries there is paucity of data regarding paediatric virological treatment failure.

## Methods

This was a retrospective cohort (1-5 yr) follow up. We studied children who started ART at the Joint Clinical Research Centre (JCRC), Kampala, Uganda under the Timetable for Regional Expansion of antiretroviral Therapy (TREAT) program between 2004 – 2009 since all children had similar free scheduled laboratory monitoring within this period.

Children who were aged 6 months to 18 years and had been initiated on ART for at least 6 months were included in the study. Those who had confirmed first line ART treatment failure and referred for second line ART were excluded plus those charts of children which had key missing information like treatment history and laboratory results (CD4 counts, viral load) were excluded.

### Study setting

The study was conducted at the JCRC Kampala, Lubowa campus, which houses the headquarters of this HIV care and research institution. It is located 10 km off Entebbe road from Kampala, the capital city of Uganda. At the time of this study, the centre cared for over 2000 HIV infected children majority of whom were perinatally infected and over 10,000 adults with respective outpatient clinics opening from Monday to Friday, 8.00a.m to 5p.m, excluding public holidays. The centre provided free services such as ART, opportunistic infections prophylactic medication, adherence counseling, routine laboratory monitoring tests, and nutritional supplementations. Among the routine laboratory monitoring tests performed were CD4 cell counts and CD4 percentages every 6 months, liver and renal function tests, plasma HIV-1(RNA) viral loads, and genotypic resistance testing when treatment failure was confirmed.

The pediatric clinic had over 1500 children under care with approximately 37 staff members namely; six pediatricians, nine nurses, four counsellors, two home visitors, one pharmacist, two pharmacy technicians, two record officers and one data assistant. Others included play room supervisors (2), phlebotomists (2) and support staff(3) with exclusion of the inpatient/ward staffs. At this clinic, treatment seeking children are recruited into care following self referral by caretakers and referral from other health centres. All HIV exposed infants awaiting DNA PCR results and those infected were/are initiated on Co- trimoxazole prophylaxis. Since most children at the clinic have/had resource constraints, most were, recruited under the PEPFAR funded programs Timetable for Regional Expansion of Anti-retroviral Therapy (TREAT).

The criteria for ART initiation and switching, as well as the ART regimens were by the WHO guidelines for ART initiation in infants and children [18]. Most children above 1 year received medication in tablet formulation with doses determined using WHO weight bands (WHO 2004,2006) [18,19]. Infants received medication largely largely in syrup form determined per kilogram body weight.

### Cohort description

We reviewed the clinic charts of patients who had been initiated on ART between Jan 2004 to September 2009, as identified from the patient care database at JCRC. This was the period of the TREAT program where all the children had similar scheduled laboratory monitoring tests like the viral loads, resistance testing and CD4 counts. The baseline was taken to be the time children were initiated on ART. The ART regimen consisted of 2 nucleoside reverse transcription inhibitors (NRTIs; lamivudine, zidovudine, stavudine and abacavir) and a non-nucleoside reverse transcription inhibitor (NNRTI, either nevirapine or efavirenz). Infants (children less than 1 year) were given syrups and older children were given tablets. The children below 3 years of age who developed Tuberculosis were put on triple NRTI regimens, to avoid the drug-drug interaction between nevirapine and rifampicin. The dosage depended on the child’s weight. In case of virologic failure, the NNRTI could be switched to the boosted protease inhibitor (PI) lopinavir/ritonavir with 2–3 NRTIs as a second-line regimen. Most of the secondline ART was chosen depending on the resistance profiles that had been done in most of the children who had failed firstline. The commonest second line ART used was combivir (lamivudine/zidovudine)didanosine(ddl) and alluvia.

In this cohort Information regarding Pre-exposure to single dose nevirapine or other antiretroviral drugs as part of a prevention of mother-to- child transmission (PMTCT) could not be entirely ascertained, because some children were orphans and brought in by distant relatives and hence the required information was not readily available.

### Laboratory assessment at the study site

Laboratory measurements included a complete blood cell count, CD4 lymphocyte count, and quantitative measurement of HIV load. Quality control assurance was done in reference to international accredited laboratories; UK National External Quality Assessment Service (UKNEQAS) and College of American Pathologists (CAP). Viral load count was done with use of Amplicor monitor standard assay, version 1.5 (Roche Molecular Systems),with a minimum detection limit of 400 copies/ml. CD4 lymphocytes were analyzed by flow cytometry (Bendict Dickson, USA). Genotypic resistance sequencing was done using in house primers. The sequences were edited using BioEdit Sequence Alignment Editor (Version 7.0.5) and analyzed using the HIV drug resistance database of Stanford University [20] for children who had a viral load greater than 2000 copies since this was the threshold beyond which mutations could be detected in the laboratory. The CD4 count and viral load were periodically done every 6 months during the routine visit to the out-patient clinic.

### Data collection

We extracted the children’s demographic and ART treatment information from their medical records at the clinic using a pre tested standardized data extraction form. This information included their gender, age at the time of assessment and ART initiation, WHO clinical staging at the start of ART, CD4 count/percentage and follow up viral loads at the time of switch were abstracted. Data regarding the use of sdNVP in PMTCT, first-line ART and the serial Weight for Height plus the six monthly follow up tests for up to five years till virological failure was first detected were extracted. History of opportunist infections, resistance mutation tests that were done before switching to second line ART tests were also recorded using the same tool. Adherence to ART was the documented adherence level by the then attending clinician during the fellow up visits. It was done by pill counts and self reports especially in the older children. An adherence greater than 95% was considered as good adherence while that less than 95% was taken as poor adherence.

Virological failure was defined as inability to achieve or maintain suppression of HIV replication to non detectable range. For this study we used virological failure as the determinant of treatment failure.

### Statistical analysis

The cumulative incidence of first-line ART failure was ascertained from the proportion of children with viral load > 2000 copies per ml at any time during follow up. Kaplan Meier survival curves plus Cox regression were used to estimate the median time to first line ART treatment failure which was the primary outcome. The Wald test was used to compare the incidence rates of ART failure in the different ART first line regimens. Factors associated with treatment failure were ascertained by comparing various variables among children who failed (cases) with those who never failed (controls) using the chi-square test for categorical data and student t test for continuous variables. Logistic regression was done to determine the factors that independently predict ART treatment failure. All social demographic and clinical characteristics (variables) were subjected to univariate analysis as (shown in Table 1 and 2). And factors whose p-valve was less than 0.02 at univariate analysis were included in multivariate analysis. The model was then built by dropping the most insignificant factor one at a time with factors whose P-valve was (<)0.05 were taken to be the factors that were independently associated with treatment failure (Table 3).

**Table 1:** A comparison of the social demographic and clinical characteristics between the cases and the controls at ART initiation of ART

**Characteristic**		**Cases (n = 240)**	**Controls (n = 461)**	**RR (95 % CI)**	**P- value**
Age	<=5 years	91 (37.9)	174 (37.7)	1.00 (0.81 – 1.24)	0.9643
	>5-10 years	61 (25.4)	139 (30.2)	0.85 (0.67 – 1.09)	0.1877
	>10 – 18 years	88 (36.7)	148 (32.1)	1.14 (0.92 – 1.41)	0.2252
Gender	Female	109 (45.4)	221 (47.9)	0.93 (0.76 – 1.15)	0.5255
	Male	131 (54.6)	240 (52.1)	1.07 (0.87 – 1.31)	0.5255
Next of kin	Parents	162 (67.5)	302 (65.5)	1.06 (0.85 – 1.32)	0.5971
	**Grandmother**	**25 (10.4)**	**81 (17.6)**	**0.65 (0.46 – 0.93)**	0.0121*
	Other	53 (22.1)	78 (16.9)	1.23 (0.97 – 1.57)	0.0961
Care taker education level	None	63 (26.5)	101 (22.3)	1.15 (0.92 – 1.45)	0.2262
	Primary	76 (31.9)	175 (38.7)	0.82 (0.66 – 1.03)	0.0783
	Secondary	58 (24.4)	102 (22.6)	1.07 (0.84 – 1.35)	0.5937
	Tertiary (not university)	26 (10.9)	43 (9.5)	1.10 (0.80 – 1.52)	0.5570
	University	15 (6.3)	31 (6.9)	0.94 (0.61 – 1.45)	0.7808
Distance from JCRC	**≤10 km**	**104 (44.3)**	**157 (35.4)**	**1.27 (1.03 – 1.56)**	0.0248*
	11 – 20 km	91 (38.7)	194 (43.8)	0.87 (0.70 – 1.08)	0.2032
	>20 km	40 (17.0)	92 (20.8)	0.85 (0.64 – 1.12)	0.2411
Past hospitalization	Yes	105 (43.8)	198 (43.2)	1.01 (0.82 – 1.25)	0.8955
	No	135 (56.2)	260 (56.8)	0.99 (0.80 – 1.21)	0.8955
Episodes of	No hospitalization	2 (2.0)	3 (1.5)	1.16 (0.39 – 3.44)	>0.999†
hospitalization	1-2 times	68 (66.6)	120 (61.9)	1.15 (0.82 – 1.61)	0.4139
	**≥ 3 times**	**32 (31.4)**	**71 (36.6)**	**0.86 (0.61 – 1.21)**	**0.3697**
Opportunistic infections	TB	51 (21.3)	90 (19.5)	1.07 (0.84 – 1.37)	0.5883
	Oral Candidiasis	39 (16.3)	64 (13.9)	1.13 (0.86 – 1.48)	0.4009
	Cryptococcal meningitis	3 (1.3)	2 (0.4)	1.76 (0.85 – 3.63)	0.3448†
	Kaposi sarcoma	2 (0.8)	5 (1.1)	0.83 (0.26 – 2.70)	>0.999†
	Pneumocystic pneumonia	3 (1.3)	5 (1.1)	1.01 (0.44 – 2.70)	>0.999†
	Oesophageal candiasis	1 (0.4)	9 (2.0)	0.29 (0.04 – 1.86)	0.1770†
	Chronic diarrhoea	4 (1.7)	18 (3.9)	0.52 (0.21 – 1.28)	0.1165†
	Bacterialmeningitis	2 (0.8)	5 (1.1)	0.83 (0.26 – 2.70)	>0.999†
HAART Regimen	**EFV**	**113(49.3)**	**278 (64.5)**	**0.67 (0.54 – 0.82)**	0.0002*
	**NVP**	**116 (50.7)**	**153 (35.5)**	**1.49 (1.21 – 1.84)**	0.0002*
Prior exposure to sdNVP	Yes	33 (13.8)	45 (9.9)	1.26 (0.95 – 1.67)	0.1253
	**No**	**207 (86.2)**	**410 (90.1)**	**0.79 (0.60 – 1.05)**	**0.1253**
Adherence	**Poor**	**116 (48.3)**	**56 (12.2)**	**2.88 (2.39 – 3.46)**	<0.0001*
	Good	124 (51.7)	405 (87.8)	0.35 (0.29 – 0.42)	<0.0001*
	I	37 (15.4)	65 (14.1)	1.07 (0.81 – 1.42)	0.6389
WHO Stage	II	106 (44.2)	198 (42.9)	1.03 (0.84 – 1.27)	0.7578
	III	85 (35.4)	163 (35.4)	1.00 (0.81 – 1.24)	0.9877
	**IV**	**12 (5.0)**	**35 (7.6)**	**0.73 (0.44 – 1.21)**	**0.1929**

CI = Confidence interval, * Significant effect, †Fisher’s exact test.

**Table 2:** Baseline CD4 counts and viral loads of study participants at ART initiation of the 701 children on ART

		**Cases**	**Controls**	
		Median (IQR)	Median (IQR)	P value*
Absolute CD4 count	Overall	241 (79–534)	313 (127–625)	0.035
	≤ 5 years	542 (273 – 1214)	728 (461 – 1129)	0.038
	>5-10 years	257 (133–474)	272 (147–382)	0.873
	>10 – 18 years	135 (50–210)	130 (44–262)	0.622
	Mean (SD)	Mean (SD)	P value	
Percentage CD4 count	Overall	15 (10.5)	16 (11.0)	0.223
	≤ 5 years	19 (10.9)	19 (9.5)	0.945
	>5-10 years	15 (8.3)	16 (12.1)	0.406
	>10 – 18 years	11 (10.0)	13 (11.0)	0.283
	Median (IQR)	Median (IQR)	P value*	
Viral load (x10 ^4^)	Overall	12.0 (2.4 – 38.6)	7.8 (2.3 - 35)	0.176
	≤ 5 years	18.8 (2.9 – 75)	17.7 (2.5 – 52.5)	0.283
	>5-10 years	4.4 (1.5 – 17.4)	6.1 (1.8 – 28.6)	0.658
	>10 – 18 years	13.1 (2.4 – 37.0)	7.6 (2.0 – 25.5)	0.183

* P value based on Wilcoxon rank-sum test.

The baseline CD4 counts, and viral loads were.

**Table 3:** Factors independently associated with treatment failure among the children on ART in the cohort at multivariate analysis

**Effect**		**OR (95% CI)**	**P- value**
CD4 counts at start of ART		0.99 (0.99-1.00)	0.027
Viral load at start of ART		0.99 (0.99-1.00)	0.402
NOK (Relationship)‡	Parents	0.9 (0.61 – 1.60)	0.965
	Grandmother	0.8 (0.42 – 1.55)	0.517
HAART Regimen	NVP	2.2 (1.40 – 3.60)	0.006*
Used sdNVP	Yes	4.2 (1.80 – 9.40)	0.001*
Adherence	Poor	10,0 (6.40 – 16.70)	<0.001*
Distance from JCRC‡‡	≤10 km	1.48 (0.88 – 2.49)	0.142
	11 – 20 km	1.21 (0.72 – 2.03)	0.470

CI = Confidence interval, * Significant effect. ‡ Reference to other caretakers.

All analyses were done using STATA version 10 (Reference: StataCorp LP, 4905 Lakeway Drive, College Station, Texas 77845, USA).

### Ethical consideration

Ethical clearance was obtained from the School of Medicine Research and Ethics committee (SOMREC), Makerere University College of Health Sciences. Waiver of consent was sought from the SOMREC and JCRC Institutional Review Board (IRB) Research and Ethics Committee (REC). Permission to carry out the study was also obtained from JCRC and the Uganda National Council of Science and Technology (UNCST).

## Results

**Figure 1:**
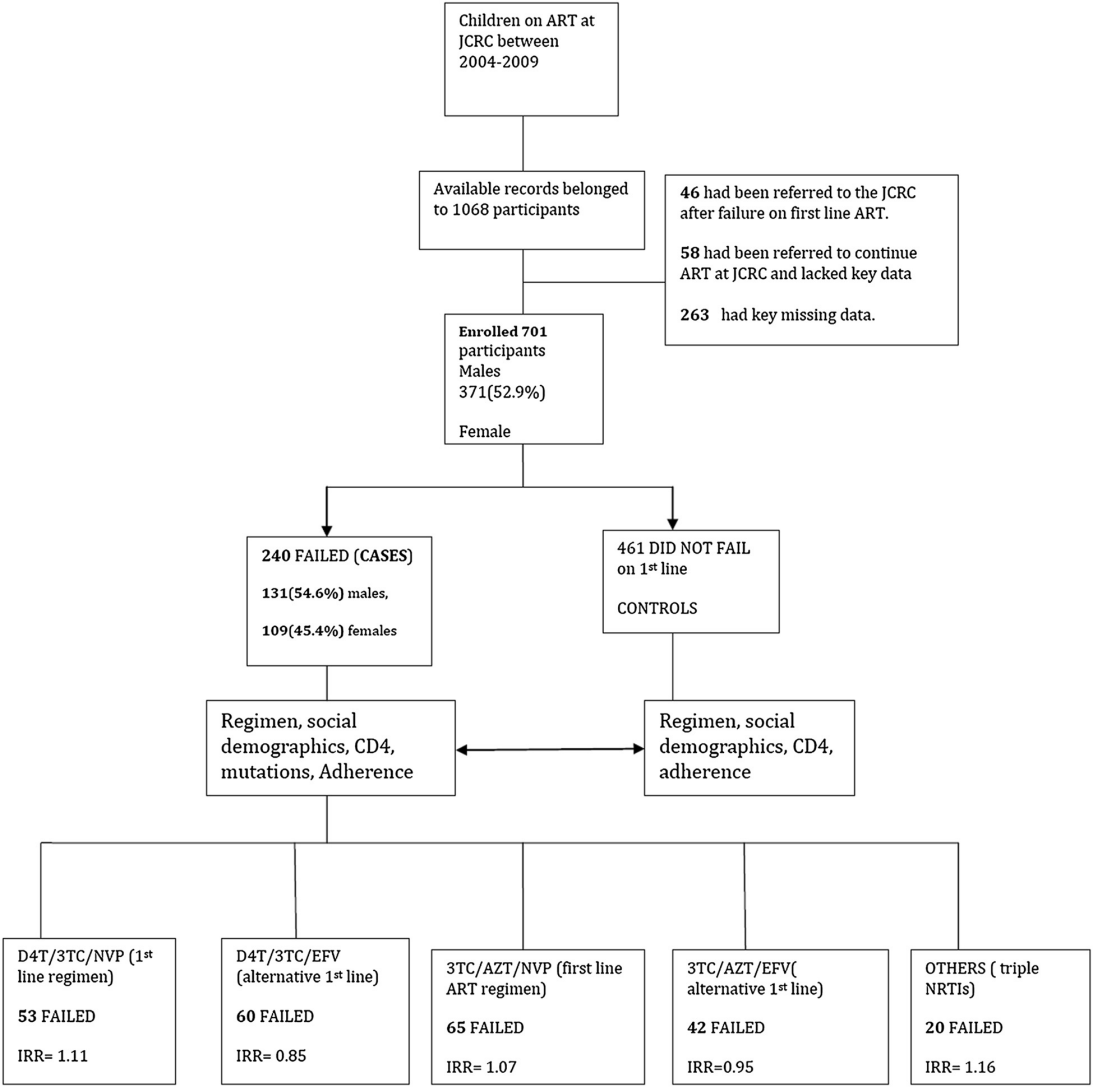
Study profile of the study participants.

We queried 1068 patients’ records from the data base who had been initiated on ART between January 1st 2004 and September 30^th^ 2009, at the JCRC paediatric out-patient clinic. Three hundred and sixty seven (367) records were excluded because some had key missing data including CD4 counts, viral loads, weight, height, resistance mutations profile results, and information on first line ART, or had been referred from other centres after documented failure on first line and others lacked baseline characteristics. The remaining 701 children’s records were included as shown in (Figure 1). Pre-exposure to single dose nevirapine or other antiretroviral drugs as part of a prevention of mother-to- child transmission (PMTCT) regimen could not be entirely ascertained in all the children though, because some children were orphan and brought in by distant relatives and hence the required information was not readily available. However 31 children had exposure to sdNVP and of those 29 were also started on neviraipne containing firstline regimen. The median baseline CD4 counts absolute and percentage at start of ART for those who failed and never failed were 241 and 313 cells,15% and 16% respectively.. Most of the children were in clinical stage two of the WHO paediatric HIV staging and were above 5 years of age. Pulmonary tuberculosis being the commonest opportunistic infection as shown in Table 4.

**Table 4:** Baseline clinical and demographic characteristics of the study participants at the time of ART initiation

**Characteristic**		**Frequency**	**%**
Age	<=5 years	265	37.80
	>5-10 years	200	28.53
	>10 – 18 years	236	33.67
Gender	Female	330	47.08
	Male	371	52.92
Next of kin	Parents	464	66.19
	Grandmother	106	15.12
	Other	131	18.69
Caretaker education level	None	164	23.77
	Primary	251	36.38
	Secondary	160	23.19
	Tertiary (not university)	69	10.00
	University	46	6.67
Past hospitalization	Yes	303	43.41
	No	395	56.59
Episodes of	0 days	5	1.69
Hospitalization	1-2 days	188	63.51
	≥ 3 days	103	34.80
Opportunistic infections	TB	141	20.11
	Oral Candidiasis	103	14.69
	CCM	5	0.71
	KS	7	1.00
	LIP	2	0.29
	PCP	8	1.14
	Esophageal	10	1.43
	Chronic_diarrhoea	22	3.14
	Bacterial meningitis	7	1.00
HAART regimen	EFV	391	59.24
	NVP	269	40.76
UsedPMTCT services	Yes	31	6.22
	No	617	88.78
Adherence	Poor (<95%)	172	24.54
	Good (>95%)	529	75.46
	I	102	14.55
WHO stage	II	304	43.37
	III	248	35.38
	IV	47	6.70

*TB*: tuberculosis, *CCM*: cryptococcal meningitis; *KS*, Kaposi’sarcoma; *LIP*, Lymphoid interstitial pnemonitis; *PCP*, pnenocystic carinii pneumonia; *EFV*, efaverenz; *NVP*, niverapine.

### Incidence of first line virological treatment failure

**Figure 2:**
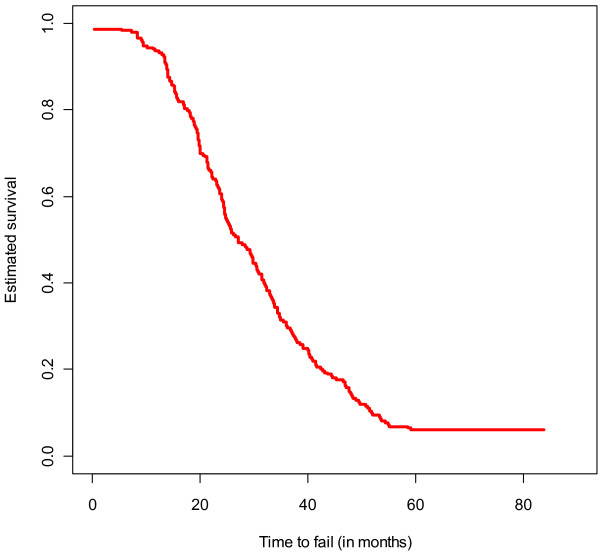
Kaplan Meier survival curve estimating the overall time to failure.

Among 701 participants, 240 (34%) cumulatively failed first-line ART. The median time to failure in months was 26.4 IQR (18.9 – 39.1). 140 (58.3%) failed in 24+ months, 80 (33.3%) failed within 12–24 months, 11(4.6%) failed within 6–12 months and only 9(3.8%) in within the first 6 months. This is also illustrated in Figure 2.

The factor that was independently associated with time to virologcal failure was poor adherence to ART as shown in Table 5 below.

**Table 5:** Factors associated with time to failure among the children who failed ART

**Effect**		**Coefficient (s.e)**	**Hazard ratio (95% CI)**	**P- value**
Age*†*	<=5 years	0.11 (0.19)	1.18 (0.82 – 1.69)	0.551
	>5-10 years	-0.05 (0.18)	0.96 (0.67 – 1.38)	0.767
NOK (Relationship) *‡*	Parents	0.13 (0.27)	0.42 (0.24 – 0.74)	0.453
	Grandmother	-0.52 (0.59)	0.86 (0.58 – 1.28)	0.053°
HAART regimen*††*	EFV	-0.11 (0.16)	0.69 (0.44 – 1.08)	0.493
Used PMTCT services	Yes	0.03 (0.23)	0.86 (0.54 – 1.39)	0.888
Adherence	Poor	-0.38 (0.14)	1.06 (0.50 – 1.10)	0.008*
Distance from JCRC	≤10 km	0.37 (0.21)	1.45 (0.97 – 2.18)	0.073
	11 – 20 km	0.36 (0.21)	1.43 (0.95 – 2.16)	0.091

† Reference to age >10 -18 yrs, ‡ Reference to other caretakers.

°Borderline significance, †† Reference to NVP, * Significant effect.

NOK next of kin, PMTCT prevention of mother to child transmission.

### Factors associated with treatment failure

Majority of the children who failed first-line ART were under five years of age (n = 91; 37.9%), male (52.5%), were under the care of parents (n = 162; 67.5%). Their caretakers attained primary education as the highest level (31.9%), as shown in Table 1. When compared to the controls, the children who failed first-line ART were more likely to live within 10 km from the ART providing institution (RR = 1.27(95% CI: 1.03-1.56), p = 0.02). Furthermore, the children who never failed were more likely to be under the care of grandmothers [(17.6% versus 10.4%), RR = 0.65 (95% CI: 0.46 -0.93), p = 0.01]. There was no statistical significance between the baseline CD4cell counts of the controls and cases (illustrated in Table 2).

Past hospitalization, opportunist Infections and WHO clinical staging were not associated with treatment failure. However, nevirapine as opposed to efavirenz as NNRTI backbone (OR = 2.2., 95% CI: 1.40 – 3.60, p = 0.001), poor adherence (OR = 10, 95% CI: 6.4 – 16.7), p < 0.0001) and prior exposure to (sdNVP) (OR = 4,2, 95% CI: 1.8 – 9,4, p = 0.002) were the significantly associated with first line ART treatment failure (Table 3). It important to note that the Viral load and CD4 at initiation of first line ART were not associated with treatment failure.

### Resistance associated mutation (RAM) in the children with treatment failure

Of the 240 children who failed first line therapy 109 had genotypic resistance profiles prior to switching to second line therapy. The commonest NNRTI RAMs was K103N (n = 59; 54%). Other common NNRTI RAMs were Y181C (n = 30; 27%), G190A (n = 26; 24%) and K101E (n = 11; 10%). The most common NRTI RAMs was the M184V (n = 89; 81%). Other common NRTI RAMs were the K70R (n = 22; 20%), T215Y (n = 36; 24%), M41L (n = 23; 21%), D67N (n = 15; 14%), K219Q/E (n = 14; 13%) and the L210W making 9% of the TAMs.

It’s worth noting that some patients had developed more than one resistance mutation at a time.

## Discussion

In this study, we have shown that the cumulative incidence of first line ART failure was 34%, with a median time to first line ART failure of 26.4 months of follow up. Varying rates of virological failure have been reported in other studies [15,21,22]. However the treatment failure rate in this study is comparable to other studies in low income countries with a high prevalence of HIV. A similar study done in rural South Africa on 101 HIV infected children reported a slightly higher virological failure rates of 38% compared to ours, with a median duration of NNRTI-based regimen of 31 months of treatment [15]. A comparable rate of 33% was reported among the Thai HIV infected children within 96 weeks of follow up [23].

In our study, patients were more likely to fail on a nevirapine NNRTI regimen compared to an efavirenz based NNRTI backbone regimen. This finding has been documented in similar studies. In the FIRST STUDY [24] by Berg-Wolf et al., the incidence of virological failure in an efavirenz based arm was 41.2 compared to 42.8 in the nevirapine arm per 100 person years after a follow up median time of 5 years. Implying that a NVP based regimen arm was associated with earlier virological failure compared to efavirenz, like our study finding.

Our study showed that treatment failure rate was more in males than the female though the difference was not statistically significant. A cohort of HIV infected children and adults performed in a similar setting (Mulago, Kampala, Uganda) reported that males were more likely to fail on ART compared to females [22]. The reason behind this sex difference is not known. Interestingly, there were more male children in our study compared to the Mulago study. Sex differences in response to ART could be biological or related to provision of care by parents. This is an area that requires further inquiry.

The factors that were independently associated with treatment failure were poor adherence to ART, use of single dose niverapine (sdNVP) in PMTCT and NVP based NNRTIs.

The critical role of adherence in the treatment of HIV has been demonstrated in many clinical trials and clinical care settings [25]. Antiretroviral therapy (ART) adherence is a strong predictor of biological (virological and immunological) and eventual clinical outcome [26-28]. This study findings are consistent with previous studies [25] and further confirms that adherence to ART is critical in the clinical, and biological outcome of ART. Our study further demonstrated that the use of single dose niverapine (sdNVP) as a measure of PMTC of HIV was strongly associated with ART first line treatment failure. This is attributed to the fact that NVP resistance occurs after the use of sdNVP as reported in many studies from all over Sub-Saharan Africa, Uganda inclusive [15,29-31]. A meta analysis to analyze the prevalence of nevirapine resistance after sdNVP was 35.7% in women and 52.6% in children [13]. The landmark HIVNET 012 trial in Uganda revealed a resistance of 46% in children and 25% in women [32]. These findings are also similar to what was reported among Ugandan children who had been exposed to sdNVP that were less likely to achieve virological suppression if initiated on a non nucleoside reverse transcriptase inhibitor (NNRTI) [33] regimen. For a long time in Uganda, NVP was used for PMTCT of HIV and has also been part of the backbone of the NNRTI regimen. It is worth noting that the study participants had been initiated on ART between 2004 to 2009 in which usage of protease Inhibitors (PI) as a backbone of the first line ART if there had been exposure to sdNVP had not been rolled out in Uganda after the recommendation from the World health Organization in 2010. It’s possible that some children could have remained on a failing regimen after exposure to sdNVP thus treatment failure.

In this study children who were initiated on a NVP based first line ART were twice as likely to fail compared to those treated with EFV containing one. A prospective longitudinal study in Thailand reported that children on a NVP backbone NNRTI were 3.7 times more likely to develop virological failure compared to efavirenz [21]. These results are also consistent with earlier findings in Kampala, Uganda [22]. The finding in this study could further be explained by the fact that efavirenz is more efficacious than nevirapine [34,35] as reported in some studies. Secondly NVP compared to EFV is commonly associated with adverse side effects and increased morbidity and mortality [36]. In the event that adverse events occur the drugs are stopped and this could contribute to poor adherence with eventual drug resistance and treatment failure.

A grandmother being the primary caretaker was less associated with treatment failure compared to other caretakers at univariate analysis. However this factor did not reach statistical significance at multivariate analysis possibly due to the small numbers of children whose primary caretaker were grandmothers. A recent Ugandan study [37] reported that children in the rural settings of Uganda had better adherence compared to their urban counterparts(91.3% vs 88.2%). Majority of grandparents in Uganda reside in rural settings and often offer more time and parental care to their grand children hence good adherence. This could explain why children whose primary caretaker was a grandmother never failed in big proportions as compared to others with different care takers.

Resistance profiles that were performed prior to switching to second line, revealed the M184V and the K103N being the commonly observed NRTI and NNRTI RAMs, respectively. TAMs occurred in 20% of the resistance profiles. This pattern of RAMs among children with virological failure on reverse transcriptase based therapy is consistent with what has been reported from studies in sub-Saharan Africa [22,38,39], Thailand. In the Thai children [21], Y181C occurred in 58% and K103N in 34% of NNRTIs RAMs. In the same study M184V/I occurred in 84%, K65R in 11%, and Q151M in 5% with TAMs occurring in 18% of the NRTIs resistance mutations. The current study reports more of the K103N as opposed to the Y181C in the NNRTIs while the proportion of M184V was consistent with the previous study. This finding was expected as most of the children at JCRC (current study) were on an efavirenz containing regimen that induces commonly the K103N as opposed to nevirapine that induces commonly the Y181C [34] which occurred in the Thai study. The NNRTIs and 3TC have a low genetic barrier to resistance. So their RAMs are the first to appear. M184V has been shown to emerge in high prevalence among children and adults failing on a 3TC-containing regimen in developed countries [40]. In Uganda, it’s been reported in children as early as 1.5 months [38] of ART. Other cross sectional studies have likewise reported a high prevalence of M184Vamong African [41,42] with mutations conferring resistance to NVP and EFV the NNRTIs occurring after 1–6 months of failure. This study showed occurrence of all the major TAMs in 20%. This finding is comparable to what Thai reports of 18% [21]. However this is in contrast to earlier reports from Uganda [20,39] that reported nearly no TAMs. Our finding could have resulted from the fact that our study had a big sample size compared to the above studies. Secondly a possibility of mother to child transmission of the RAMs since baseline resistance testing prior to ART initiation was never done cannot be ruled out. However this study highlights the fact that the thymine analogue mutations are present in our setting and their presence will compromise second line ART options.

### Study strength and limitations

We studied a relatively large number of HIV infected children on ART to examine the incidence of and median time to first line ART failure compared to similar studies. We however acknowledge some limitations in our study. Being a retrospective nested case control, we could not control for some of the possible confounders. Secondly baseline resistance testing was not done, so the exact impact of primary drug resistance prior to ART initiation and the accumulation of RAMs is unknown.

## Conclusion and recommendations

In a low resource setting where nevirapine still forms a backbone of first-line ART like Uganda, our findings suggest that one in three children on first-line ART are likely to develop virological treatment failure after the first 24 months of therapy.

We recommend that, efavirenz as opposed to nevirapine be used as first-line non-nucleoside backbone. Furthermore that adherence to ART should be emphasized at all levels of care in paediatric ART service providers to prevent the development of treatment failure.

## Abbreviations

RS: Robert Sebunya; VM: Victor Musiime; SBK: Sabrina Bakeera Kitaka; GN: Grace Ndeezi; JCRC: Joint clinical resarch centre; ART: Antiretroviral therapy; PMTC: Prevention of mother to child transmission; RAMs: Resistance associated mutations; TAMs: Thymidine analogue mutations; NNRTIs: Non nucleoside reverse transcriptase inhibitors; NRTIs: Nucleotide reverse transcriptase inhibitors; NVP: Nevirapine; EFV: Efavirenz.

## Competing interests

The authors declare that they have no competing interests.

## Authors’ contributions

RS; designed the study, collected data, interpreted study findings and wrote up the primary draft manuscript. GN, VM: Helped with the interpretation of the study findings, reviewed and assisted with the critical revision of the manuscript before submission.SBK: reviewed and commented on first draft manuscript before submission. VM was one of the Paediatricians that offered care for the children at the study site. All authors read and approved the final manuscript.
